# Supplementary material for: Chlorate Contamination in Commercial Growth Media as a Source of Phenotypic Heterogeneity within Bacterial Populations
Source: Microbiol Spectr. 2023 Feb 8;11(2):e04991-22. doi: 10.1128/spectrum.04991-22 (PMC10100951; doi:10.1128/spectrum.04991-22)
Supplement: Supplemental file 1 — Fig. S1 and S2. Download spectrum.04991-22-s0001.pdf, PDF file, 0.1 MB [file spectrum.04991-22-s0001.pdf]

Supplemental material

Chlorate contamination in commercial growth media as a source of phenotypic heterogeneity within bacterial populations

Maxence S. Vincent[1], Alexandra Vergnes[1], Benjamin Ezraty[1] *

[1] Aix-Marseille Université, CNRS, Laboratoire de Chimie Bactérienne, Institut de Microbiologie de la Méditerranée, Marseille, France.

* Corresponding author: ezraty@imm.cnrs.fr

Supplementary Figure S1

Supplementary Figure S2

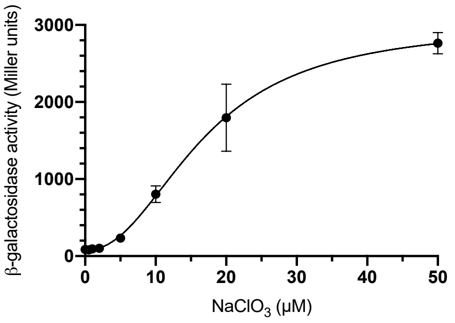

Supplementary Figure S1: Determination of the *PhiuH-lacZ* reporter sensitivity

ß-galactosidase activities of *PhiuH-lacZ* (strain CH184) as a function of the concentration of chlorate.

A

	Product	Company	Reference	Batch
**1**	Bacto Casamino Acids	BD	223050	6266538
**0**	Agarose	Sigma	A9539	102490311
**7**	Pastagar A	Biorad	3564985	64391006
**8**	Bacto Agar	BD	214010	1208830
**9**	Agar Agar	Euromedex	1330	2001000011
**10**	Agar Bacteriological	Oxoid	LP0011	2113685-02

B

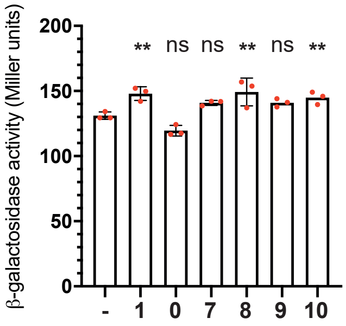

Supplementary Figure S2: *PhiuH-lacZ* expression during aerobic growth

(A) A number was attributed to the different compounds used in panel B to facilitate figure interpretation. (B) ß-galactosidase activities of *PhiuH-lacZ* (strain CH184) were measured after overnight aerobic growth at 37 °C in LB (-) supplemented or not with 0.4% BD Bacto CASA (1), agarose (0) or different agar powders (7, 8, 9, 10). ** indicates a p-value < 0.01 using a Dunnett's multiple comparison test.
